# Identification of markers predicting clinical course in patients with Behcet disease by combination of machine learning and unbiased clustering analysis

**DOI:** 10.1007/s00417-025-06850-5

**Published:** 2025-05-06

**Authors:** Kinya Tsubota, Yoshihiko Usui, Hiroyuki Shimizu, Hiroshi Goto

**Affiliations:** Department of Ophthalmology, Tokyo Medical University, 6-7-1 Nishishinjuku, Shinjuku-Ku, Tokyo, 160-0023 Japan

**Keywords:** Behçet’s disease, Machine learning, TNF inhibitor, Unsupervised hierarchical clustering, Monocyte percentage, Neutrophil/lymphocyte ratio

## Abstract

**Purpose:**

Behçet's disease (BD) is a multisystem inflammatory disorder with diverse clinical manifestations. Identifying biomarkers predictive of clinical outcomes, such as tumor necrosis factor (TNF) inhibitor initiation and ocular inflammatory attack frequency, is critical for improving management. This study aimed to identify biomarkers predicting the clinical course of BD using peripheral blood test data and unbiased clustering combined with machine learning.

**Methods:**

A retrospective cohort study of 238 BD patients diagnosed at Tokyo Medical University Hospital (2004–2020) was conducted. Unsupervised hierarchical clustering was applied to peripheral blood data, dividing patients into distinct groups. Machine learning techniques were used to explore biomarkers predicting the clinical course.

**Results:**

Cluster analysis identified four groups: Group A (low C-reactive protein), Group B (high angiotensin-converting enzyme), Group C (high anti-streptolysin O), and Group D (low neutrophil count). Group C had a higher rate of TNF inhibitor initiation (47%, p = 0.04), while Group D had fewer ocular inflammation attacks per year (1.4, p = 0.04). Logistic regression analysis identified red blood cell count (p < 0.01) and monocyte percentage (p = 0.02) as predictive biomarkers for TNF inhibitor initiation. Machine learning further confirmed mean corpuscular hemoglobin concentration (MCHC) as a significant predictor of TNF inhibitor initiation. Additionally, multiple regression analysis identified the neutrophil/lymphocyte ratio as a predictor of the number of inflammatory attacks per year (p = 0.02).

**Conclusions:**

Unsupervised clustering of blood test data identified distinct BD clinical phenotypes. Monocyte percentage may predict TNF inhibitor initiation, while neutrophil/lymphocyte ratio may predict ocular inflammation frequency, highlighting pathophysiologic heterogeneity in BD.

**Supplementary Information:**

The online version contains supplementary material available at 10.1007/s00417-025-06850-5.

## Abstract (highlights)

**Key messages:**

*****What is known***:**

- Behçet’s disease (BD) is an inflammatory disorder with diverse clinical manifestations, and early initiation of tumor necrosis factor (TNF) inhibitors is effective in improving visual prognosis.

*****What is new***:**

- Unbiased clustering analysis divided patients into four groups: low C-reactive protein (CRP), high angiotensin-converting enzyme (ACE), high anti-streptolysin O (ASLO), and low neutrophil count. The ASLO-high group showed a significantly higher rate of TNF inhibitor initiation, and ocular inflammatory attacks per year was significantly lower in the low neutrophil count group.
- Machine learning and statistics analysis identified mean corpuscular hemoglobin concentration (MCHC) and monocyte percentage as predictors of TNF inhibitor initiation.
- The neutrophil-to-lymphocyte ratio was also identified as a significant predictor of the frequency of ocular inflammatory attacks.

**Supplementary Information:**

The online version contains supplementary material available at 10.1007/s00417-025-06850-5.

## Introduction

Many systemic diseases are etiologies of uveitis [1]. Behçet’s disease (BD) constitutes a relatively large proportion of all uveitis cases in Japan [1–4]. In many countries, BD also ranks relatively high among uveitis in epidemiological surveys [5–9]. BD may cause worsening of visual acuity due to repeated ocular inflammatory attacks. While its precise etiology remains unclear, BD is characterized by a complex interplay of genetic, environmental, and immunological factors [10]. Epidemiologically, the disease demonstrates a distinct geographical distribution, with higher prevalence observed along the historical"Silk Road,"encompassing regions such as East Asia, the Middle East, and the Mediterranean basin [11]. Clinically, BD manifests with a wide array of symptoms, including recurrent oral and genital ulcers, skin lesions, uveitis, and systemic involvement such as vascular, gastrointestinal, and neurological complications [11]. Among these, uveitis is a major cause of morbidity, often leading to visual acuity loss due to recurrent ocular inflammatory attacks. The advent of tumor necrosis factor (TNF) inhibitors, such as infliximab and adalimumab, has revolutionized the management of BD-related uveitis [12–16]. These agents have demonstrated significant efficacy in controlling ocular inflammation and preventing vision loss, especially in patient’s refractory to conventional immunosuppressive therapies [12–16].

Although visual outcome was improved by initiation of tumor necrosis factors (TNF) inhibitors such as infliximab and adalimumab [12–16], some patients have poor visual acuity despite using TNF inhibitors. On the contrary, some patients never have ocular inflammatory attack even without treatment with TNF inhibitors. Furthermore, BD is known to cause a diversity of symptoms such as skin and mucosal lesions, vascular lesions, uveitis, gastrointestinal lesions, and neurological lesions, and cluster analysis of BD phenotypes has recently been reported [17, 18]. The manifestation and severity of BD involvement may exhibit variability and heterogeneity over time, thereby necessitating a tailored therapeutic approach. Some patients do not achieve visual acuity improvement by TNF inhibitors because severe intraocular inflammation can cause irreversible damage to retina and optic nerve [12]. Therefore, appropriate treatments including early initiation of TNF inhibitors are required to anticipate ocular inflammatory attacks before irreversible damage occurs. However, biomarkers for predicting the clinical course of BD are not clearly identified.

Peripheral blood tests are sometimes performed for the diagnosis of uveitis. For example, serum soluble interleukin-2 receptor is a biomarker for diagnosing sarcoidosis, high white blood cell count is a biomarker of bacterial infection, and neutrophil to lymphocyte ratio is elevated in patients with BD [19–21]. Recently, artificial intelligence (AI) technology has been utilized in predicting the outcome and diagnosis of various diseases in the field of ophthalmology [22–25]. Machine learning is a branch of AI, which finds hidden data by using a computer to learn iteratively from data sets and apply the learned results to new data to predict the results according to patterns. We have reported that machine learning using peripheral blood data is potentially useful for searching predictive biomarkers in IgG4-related ophthalmic disease and primary vitreoretinal lymphoma [26, 27]. However, no attempt has been made so far to apply machine learning to peripheral blood test data for predicting the outcome of BD.

The purpose of this study was to perform hierarchical cluster analysis using routine peripheral blood test data and compare the clinical course between clusters to determine whether peripheral blood tests alone can predict the clinical course of BD, including the need to initiate TNF inhibitors and the frequency of ocular inflammatory attacks. In addition, machine learning and statistical techniques were applied to identify biomarkers that predict the clinical course of disease. By achieving these objectives, this study seeks to improve treatment strategies, such as the early introduction of biologics before severe intraocular inflammation causes irreversible damage to the retina and optic nerve.

## Materials and methods

### Subjects

We conducted a retrospective, noninterventional, single institution, observational study. We reviewed the medical records at the Department of Ophthalmology, Tokyo Medical University Hospital between 2004 and 2020, and identified 238 patients with BD. Among these patients, we excluded 62 patients because of no peripheral blood test results and included the remaining 176 patients with BD in this analysis. BD was diagnosed according to the diagnosis criteria of the Japanese Ministry of Health, Labor, and Welfare Designated Disease Study Group [28]. Routine peripheral blood tests for diagnosis were performed at the first visit to our hospital. The details of prior treatments received at the previous medical institutions before the initial visit are summarized in Supplemental Table S1. Among the patients analyzed, 24 (14%) had no prior treatment, 50 (28%) had received only topical treatment, 65 (37%) had been taking combination systemic therapy with medications or supplements due to systemic complications, and 37 (21%) had incomplete treatment records that could not be verified. The following clinical data were extracted from the medical records of each patient: gender, age, best corrected visual acuity (BCVA) at first visit, BCVA at final visit, Behçet's disease ocular attack score 24 (BOS24 score) [29], macular edema prevalence, secondary glaucoma prevalence, and rate of TNF inhibitor initiation.

### Unsupervised hierarchical clustering analysis

Unsupervised hierarchical clustering analysis was performed using the following continuous variables which are routinely examined for the patients with uveitis: peripheral white blood cell (WBC) count, peripheral red blood cell (RBC) count, percent lymphocyte (Lym), Hematocrit (Ht), mean corpuscular volume (MCV), mean corpuscular hemoglobin (MCH), mean corpuscular hemoglobin concentration (MCHC), percent eosinophil (Eo), percent neutrophils (Neut), percent basophils (Baso), percent monocytes (Mono), neutrophil count, reticulocyte (Ret), red blood cell distribution width: coefficient of variation (RDW-CV), RDW: standard deviation (RDW-SD), PLT: mean platelet volume (PLT-MPV), PLT: platelet distribution width (PLT-PDW), PLT: large cell ratio (PLT-large cell), erythrocyte sedimentation rate (ESR), platelet (PLT) count, serum total protein (TP), aspartate aminotransferase (AST), alanine aminotransferase (ALT), γ-glutamyl transferase (γ-GTP), lactate dehydrogenase (LDH), alkaline phosphatase (ALP), total bilirubin (T-BIL), blood urea nitrogen (BUN), urinary acid (UA), creatinine (Cre), C-reactive protein (CRP), creatinine (Cre), sodium (Na), chloride (Cl), potassium (K), calcium (Ca), glucose (Glu), total cholesterol (Chol), CRP, 50% hemolytic complement activity (CH50), rheumatoid factor (RF), anti-streptolysin-O (ASLO), immunoglobulin G (IgG), immunoglobulin A (IgA), immunoglobulin E (IgE), immunoglobulin M (IgM) and angiotensin converting enzyme (ACE).

We divided the patients into 4 groups by unbiased clustering analysis. Both normalization and standardization are performed because the units are different for each blood collection item and there is a possibility that outlier cases may exist. However, outlier cases were not excluded from the analysis, considering the possibility that they may represent unusual events or unique groups [30]. Euclidean Distance is used to calculate distances, and Ward's method is used to calculate distances between clusters, according to previous report [26, 27]. We then compared the 4 groups for the following variables: BCVA at first visit, BCVA at final visit, BOSS24 score [29], frequency of recurrence, macular edema prevalence, secondary glaucoma prevalence, and rate of TNF inhibitor initiation. Receiver operating characteristic (ROC) curves were generated to predict TNF inhibitor initiation in the future. ROC curves were also generated using multiple important factors selected by machine learning or logistic regression analysis, and the areas under the ROC curve (AUC) were compared. Machine learning using random forest was performed by Boruta multivariate analysis (https://notabug.org/mbq/Boruta/) according to previous reports [31]. The Boruta feature selection was based on package “Boruta” in R. The Boruta feature selection was based on the package “Boruta” in R. The Boruta algorithm is based on random forest to identify the most important features. Boruta iteratively evaluates feature importance using a shadow feature matrix to identify significant predictors [32]. This approach ensures robust feature selection while avoiding overfitting by leveraging the OOB error rate. Our model achieved a minimum OOB error rate with Ntrees = 500, ensuring optimal convergence and generalization.

### Statistical analysis

Kruskal–Wallis test, Steel–Dwass test and chi-squared test were used to compare clinical findings among 4 groups divided by unbiased clustering analysis. Logistic regression analysis was performed using peripheral blood test data as independent variables and TNF inhibitor initiation as the dependent variable. Clinical risk factors associated with frequency of ocular inflammatory attacks were analyzed by multiple regression analysis. A p value less than 0.05 was considered statistically significant. All analyses were performed using commercial statistical analysis software (BellCurve for Excel, Social Survey Research Information Co. Ltd., Japan; JMP, SAS Institute, Ltd., Japan).

## Results

### Clustering analysis

Patient demographics are shown in Table 1. The 176 patients consisted of 103 males and 73 females, with an average onset age of 38.4 ± 14.5 (mean ± SD) years. BCVA was 0.49 ± 0.65 logMAR at the first visit, and 0.28 ± 0.58 logMAR at the last visit. Mean number of ocular inflammatory attacks per year was 3.8 ± 2.8, and mean BOS24 score was 5.5 ± 3.5. BCVA improved from the first visit to the last visit in many patients. There were 62 cases of macular edema (35%), 35 cases of glaucoma (19%), and 44 cases of TNF inhibitor initiation (25%).

**Table 1:** Patients demographics

Characteristics	No. (%) or average (± SD)
Number of patients, n	176
Sex: male/female, n	103/73
Age, years	38.4 ± 14.5
BCVA at 1 st visit (logMAR)	0.49 ± 0.65
BCVA at final visit (logMAR)	0.28 ± 0.58
Mean number of ocular inflammation attacks per year	3.8 ± 2.8
Mean BOS24 score	5.5 ± 3.5
Prevalence of macular edema, n (%)	62 (35%)
Prevalence of glaucoma, n (%)	35 (19%)
TNF inhibitors initiation, n (%)	44 (25%)

BCVA best corrected visual acuity, BOS24 Behcet's disease ocular attack score 24, TNF tumor necrosis factor

**Fig. 1:**
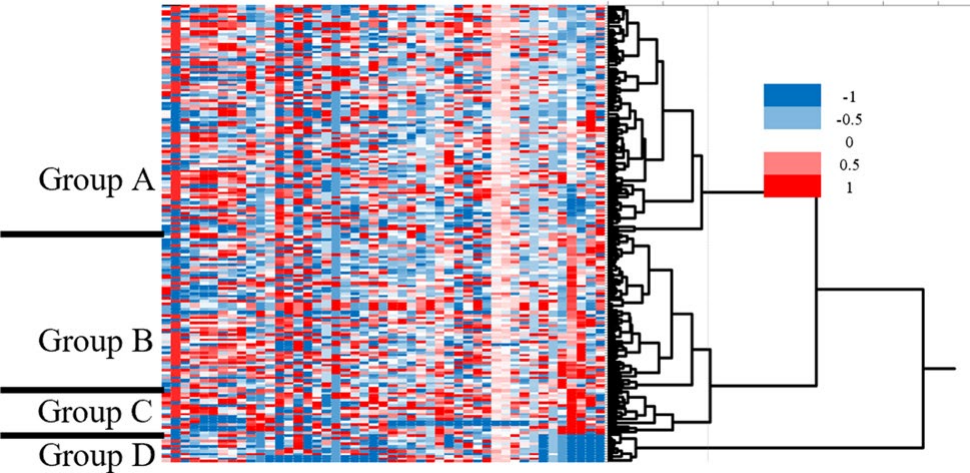
Unsupervised hierarchical clustering analysis using the data of 47 peripheral blood tests. The vertical axis represents individual patients, while the horizontal axis shows different laboratory parameters. The dendrogram on the right indicates hierarchical relationship among patients, highlighting clusters with similar laboratory test profiles. The heatmap color scheme represents the relative value of each parameter, where higher values are displayed in shades of red, and lower values in shades of blue. Clustering analysis divides patients with Bechet’s disease into 4 groups. Group A, patients with low C-reactive protein; Group B, patients with high angiotensin-converting enzyme; Group C, patients with high anti-streptolysin O; Group D, patients with low neutrophil count

First, we applied unbiased clustering analysis to obtain a global overview of laboratory test data at first visit (Fig. 1). Using unbiased clustering analysis, patients were divided into 4 groups. Group A was the largest cluster (n = 88; 50% of subjects) consisting of patients who had low CRP. Sixty subjects (34%) were grouped into group B consisting of patients with high ACE. Seventeen subjects (10%) were grouped into group C consisting of patients with high ASLO. Group D was the smallest cluster (n = 11; 6% of subjects) consisting of patients with low Neut count. Next, we compared the clinical features between groups A, B, C and D, as shown in Table 2. Although BCVA at first visit was significantly worse in group C (with higher ASLO) compared with the other groups (p = 0.04), BCVA at final visit did not differ among the four groups (p = 0.28). The mean number of ocular inflammatory attacks per year was significantly lower in group D (with lower Neut count) compared with the other groups (p = 0.04), and mean BOS24 score was apparently lower in group D, although the difference was not significant (p = 0.18). Comorbidity rates for macular edema and glaucoma were similar among the four groups (p = 0.43 and 0.80, respectively). The rate of TNF inhibitor initiation was significantly higher in group C (with higher ASLO) compared with the other groups (p = 0.04), possibly due to poor BCVA at the first visit.

**Table 2:** Clinical findings in four groups classified by unbiased clustering analysis

Characteristics	Group A	Group B	Group C	Group D	p value
BCVA at 1 st visit (logMAR)	0.59	0.00	1.00	0.29	0.04
BCVA at final visit (logMAR)	0.38	0.00	0.00	0.26	0.28
Mean number of ocular inflammation attacks per year	3.8	4.0	4.0	1.4	0.04
Mean BOS24 score	5.8	6.0	7.0	2.7	0.18
Prevalence of macular edema, n (%)	36 (41%)	17 (28%)	5 (29%)	4 (36%)	0.43
Prevalence of glaucoma, n (%)	18 (20%)	13 (22%)	3 (18%)	1 (9%)	0.80
TNF inhibitor initiation, n (%)	24 (27%)	11 (18%)	8 (47%)	1 (9%)	0.04

BCVA best corrected visual acuity, BOS24 Behcet's disease ocular attack score 24, TNF tumor necrosis factor

### Logistic regression analysis and Boruta analysis

**Fig. 2:**
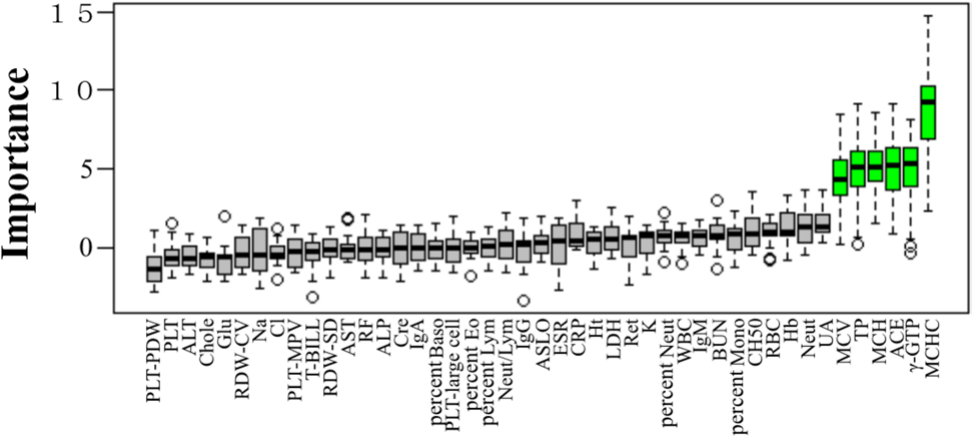
Machine learning analysis using random forest by Boruta analysis for prediction of TNF inhibitor initiation. Green box denotes a feature confirmed to be important; gray box denotes unimportant. MCHC, γ-GTP, ACE, MCH, TP and MCV are more important for prediction of TNF inhibitor initiation. MCHC, mean corpuscular hemoglobin concentration; γ-GTP, γ-glutamyl transferase; ACE, angiotensin converting enzyme; MCH, mean corpuscular hemoglobin; TP, total protein; MCV, mean corpuscular volume

We then performed a logistic regression analysis and Boruta analysis to search for clinical parameters such as routine peripheral blood test data associated with TNF inhibitor initiation (Table 3, Fig. 2). In logistic regression analysis, RBC count (odds ratio: 2.5 × 109, 95% confidence interval [CI]: 1.11 × 103–5.76 × 1017, p < 0.01) and percent Mono (odds ratio: 17.2, 95% CI: 1.62–182.23, p = 0.02) had significantly high odds ratios. Although the odds ratios were not high, a significant association was found in Chole, BUN and ASLO (p = 0.03, 0.03 and 0.03, respectively). Boruta analysis identified MCHC (importance score: 8.5), γ-GTP (importance score: 5.1), ACE (importance score: 5.0), MCH (importance score: 4.9), TP (importance score: 4.8) and MCV (importance score: 4.2) as important peripheral blood findings for predicting initiation of TNF inhibitor (Fig. 2).

**Table 3:** Logistic regression analysis of clinical risk factors associated with initiation of TNF inhibitors

Characteristics	Odds ratio	95% CI		P value	Characteristics	Odds ratio	95% CI		P value
		Lower	Upper				Lower	Upper	
RBC	2.5 × 10^9^	1.11 × 10^3^	5.76 × 10^17^	< 0.01	IgG	1.0	1.00	1.00	0.40
Percent Mono	17.2	1.62	182.23	0.02	IgM	1.0	0.99	1.01	0.70
MCH	7.9	0.05	1.21 × 10^3^	0.42	IgA	1.0	0.99	1.00	0.41
K	3.4	0.98	11.97	0.05	CH50	1.0	0.97	1.03	0.81
T-BIL	2.7	0.43	17.12	0.29	Glu	1.0	0.97	1.02	0.55
MCHC	2.1	0.02	181.61	0.75	LDH	1.0	0.98	1.00	0.02
MCV	1.8	0.27	11.46	0.55	PLT-large	1.0	0.86	1.14	0.89
Percent Baso	1.6	0.00	4.59 × 10^3^	0.90	ALT	1.0	0.94	1.02	0.40
PLT-MPV	1.6	0.90	2.69	0.12	AST	1.0	0.90	1.06	0.56
Neut	1.4	0.89	2.22	0.15	ESR	1.0	0.93	0.99	0.01
UA	1.3	0.93	1.82	0.13	RDW-SD	1.0	0.85	1.07	0.43
BUN	1.1	1.01	1.26	0.03	PLT-PDW	0.9	0.61	1.45	0.80
Na	1.1	0.92	1.39	0.24	ACE	0.9	0.80	0.96	< 0.01
WBC	1.1	0.91	1.28	0.36	Cl	0.8	0.68	1.06	0.14
Ret	1.0	0.97	1.09	0.36	RDW-CV	0.8	0.58	1.14	0.23
Percent Neut	1.0	0.99	1.07	0.20	CRP	0.7	0.43	1.15	0.17
Cre	1.0	0.04	28.54	0.99	TP	0.7	0.26	1.91	0.49
γ-GTP	1.0	1.00	1.02	0.13	Neut/lymph ratio	0.7	0.36	1.28	0.23
RF	1.0	0.98	1.04	0.56	Percent Lym	0.5	0.18	1.62	0.27
Chol	1.0	1.00	1.02	0.03	Ht	0.4	0.01	15.06	0.64
ASLO	1.0	1.00	1.01	0.03	Percent Eo	0.1	0.0.1 × 10^–1^	1.53	0.09
AL-P	1.0	1.00	1.01	0.08	Hb	> 0.01	0.02 × 10^–5^	177.39	0.33
PLT	1.0	1.00	1.01	0.72					

WBC white blood cell count, RBC red blood cell count, Lym lymphocyte, Ht Hematocrit, MCV mean corpuscular volume, MCH mean corpuscular hemoglobin, MCHC mean corpuscular hemoglobin concentration, Eo eosinophil, Neut neutrophil, Baso basophils, Mono monocytes, Ret reticulocyte, RDW-CV red blood cell distribution width coefficient of variation, RDW-SD RDW standard deviation, PLT platelet, PLT-MPV platelet mean platelet volume, PLT-PDW platelet distribution width, PLT-large platelet large cell ratio, ESR erythrocyte sedimentation rate, TP total protein, AST aspartate aminotransferase, ALT alanine aminotransferase, γ-GTP γ-glutamyltransferase, LDH lactate dehydrogenase, ALP alkaline phosphatase, T-BIL total bilirubin, BUN blood urea nitrogen, UA urinary acid, Cre creatinine, CRP C-reactive protein, Cre creatinine, Na sodium, Cl chloride, K potassium, Ca calcium, Glu glucose, Chol total cholesterol, CH50 50% hemolytic complement activity, RF rheumatoid factor, ASLO antistreptolysin-O, IgG immunoglobulin G, IgA immunoglobulin A, IgE immunoglobulin E, IgM immunoglobulin M, ACE angiotensin converting enzyme

**Fig. 3:**
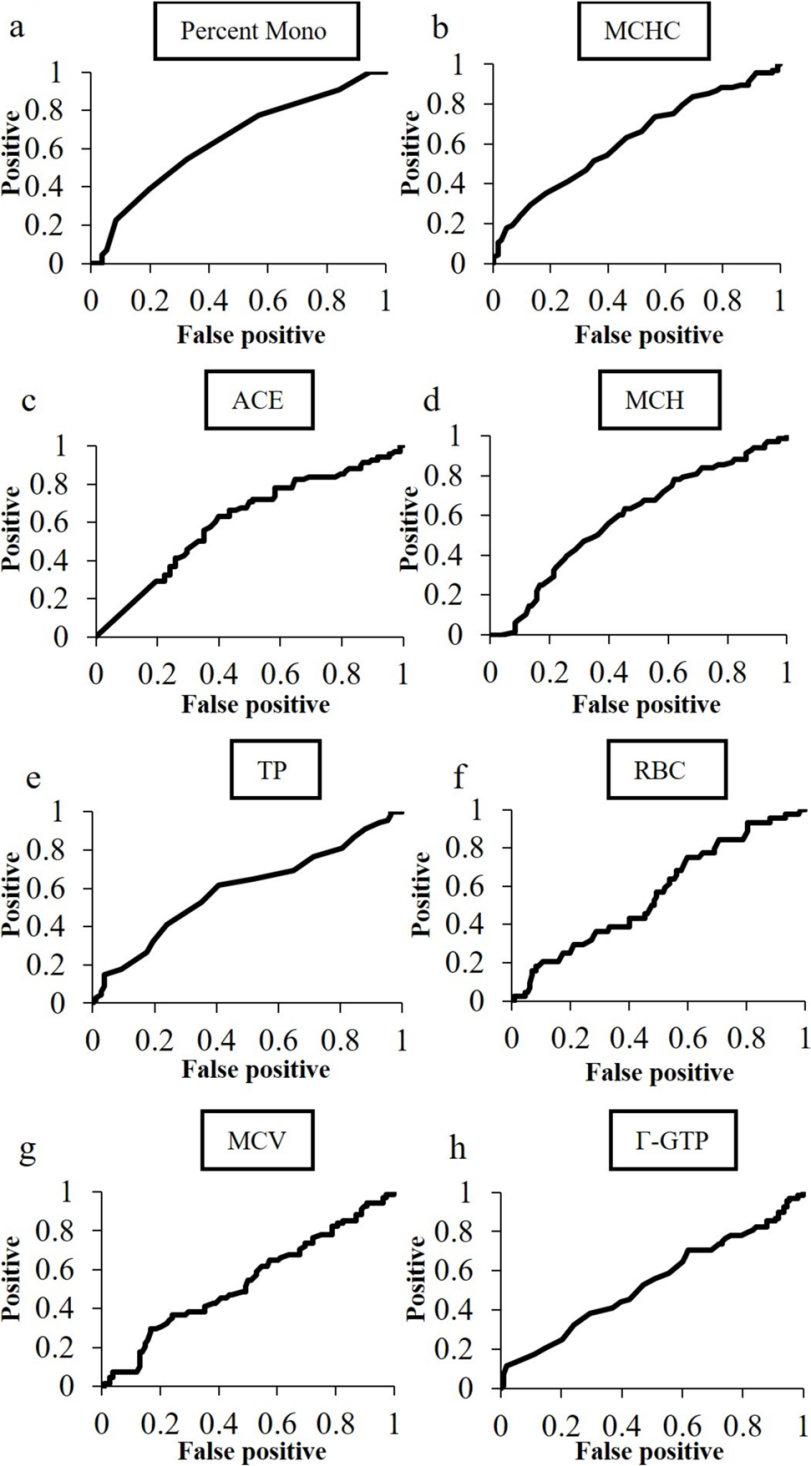
ROC curves for predicting initiation of TNF inhibitor using important peripheral blood test data. Percent Mono had the greatest AUC (0.64) and the second greatest AUC was MCHC (0.62). Mono, monocytes; MCHC, mean corpuscular hemoglobin concentration; ACE, angiotensin converting enzyme; MCH, mean corpuscular hemoglobin; TP, total protein; RBC, red blood cell count; MCV, mean corpuscular volume; γ-GTP, γ-glutamyl transferase

We plotted receiver operating characteristic (ROC) curves to investigate which laboratory finding among RBC count, percent Mono, MCHC, γ-GTP, ACE, MCH, TP and MCV is most suitable for predicting TNF inhibitor initiation (Fig. 3, Table 4). The areas under the ROC curves for percent Mono, MCHC, ACE, MCH, TP, RBC count, MCV and γ-GTP were 0.64, 0.62, 0.61, 0.59, 0.59, 0.56, 0.54 and 0.54, respectively. These results indicate that percent Mono is most suitable for the prediction of TNF inhibitor initiation in the future.

**Table 4:** Cutoff values obtained from ROC curves

Marker	AUC	Odds ratio	Cutoff value		p value
Percent Mono	0.64	2.48	0.5	%	0.03
MCHC	0.62	2.00	33.8	%	< 0.01
ACE	0.61	2.60	8.6	U/L	0.01
MCH	0.59	2.07	30.4	pg	0.04
TP	0.59	2.35	7.3	g/dL	0.04
RBC count	0.56	1.67	4500 × 10^4^	/µL	0.18
MCV	0.54	1.34	89.1	fL	0.42
γ-GTP	0.54	1.26	20	U/L	0.43

ROC receiver operating characteristic, AUC area under the ROC curve, Mono monocyte, MCHC mean corpuscular hemoglobin concentration, ACE angiotensin converting enzyme, MCH mean corpuscular hemoglobin, TP total protein, RBC red blood cell, MCV mean corpuscular volume, γ-GTP γ-glutamyl transferase

Finally, we performed a multiple regression analysis to search for routine peripheral blood test data that are associated with the number of ocular inflammatory attacks per year (Table 5). Neut/Lymph ratio (partial regression coefficient: 0.4, 95% CI: 0.06–0.77, p = 0.02) and RDW-CV (partial regression coefficient: 0.4, 95% CI: 0.11–0.72, p = 0.01) had significantly high partial regression coefficient. The above results thus indicate that Neut/Lymph ratio and RDW-CV are good predictors of the frequency of recent ocular inflammatory attacks, and percent Mono is a good predictor for initiation of TNF inhibitor in the future.

**Table 5:** Multiple regression analysis of clinical risk factors associated with the number of ocular inflammatory attacks per year

Characteristics	Partial regression coefficient	95% CI		P value	Characteristics	Partial regression coefficient	95% CI		P value
		Lower	Upper				Lower	Upper	
MCHC	2.0	−0.60	4.59	0.13	ALP	< 0.1	0.00	0.00	0.36
Lym	0.9	−0.44	2.34	0.18	PLT	< 0.1	−0.01	0.00	0.15
Ht	0.9	−1.60	3.42	0.47	CH50	< 0.1	−0.02	0.01	0.73
Neut/lymph ratio	0.4	0.06	0.77	0.02	Glu	< 0.1	−0.02	0.01	0.70
RDW-CV	0.4	0.11	0.72	0.01	LDH	< 0.1	−0.01	0.00	0.38
TP	0.3	−0.08	0.78	0.11	IgM	< 0.1	−0.01	0.00	0.03
WBC	0.2	−0.59	1.06	0.58	RF	< 0.1	−0.03	0.01	0.41
MCV	0.2	−1.10	1.53	0.75	RDW-SD	< 0.1	−0.10	0.07	0.76
Percent Eo	0.1	−2.21	2.45	0.92	ALT	< 0.1	−0.04	0.01	0.24
Percent Mono	0.1	−1.78	2.01	0.90	Ret	< 0.1	−0.08	0.02	0.23
PLT-large	0.1	−0.04	0.17	0.23	Cl	< 0.1	−0.19	0.08	0.43
Na	< 0.1	−0.06	0.16	0.38	Ua	< 0.1	−0.27	0.13	0.48
ESR	< 0.1	0.00	0.05	0.05	PLT-PDW	< 0.1	−0.42	0.26	0.63
ACE	< 0.1	−0.04	0.08	0.49	PLT-MPV	< 0.1	−0.71	0.13	0.17
Percent Neut	< 0.1	−0.05	0.08	0.68	K	< 0.1	−1.34	0.36	0.26
BUN	< 0.1	−0.07	0.09	0.83	Neut	< 0.1	−1.38	0.16	0.12
AST	< 0.1	−0.05	0.06	0.86	T-BIL	< 0.1	−2.03	0.53	0.25
CRP	< 0.1	−0.19	0.20	0.96	Cre	< 0.1	−3.17	1.47	0.47
γ-GTP	< 0.1	0.00	0.01	0.23	MCH	< 0.1	−4.29	2.43	0.58
Chol	< 0.1	0.00	0.01	0.20	RBC	< 0.1	−11.70	9.24	0.82
IgA	< 0.1	0.00	0.00	0.67	Hb	< 0.1	−8.28	4.64	0.58
IgG	< 0.1	0.00	0.00	0.47	Percent Baso	< 0.1	−11.63	0.98	0.10
ASLO	< 0.1	0.00	0.00	0.41					

WBC white blood cell count, RBC peripheral red blood cell count, Lym lymphocyte, Ht Hematocrit, MCV mean corpuscular volume, MCH mean corpuscular hemoglobin, MCHC mean corpuscular hemoglobin concentration, Eo eosinophil, Neut neutrophil, Baso basophils, Mono monocytes, Ret reticulocyte, RDW-CV red blood cell distribution width coefficient of variation, RDW-SD red cell distribution width: standard deviation, PLT platelet, PLT-MPV platelet mean platelet volume, PLT-PDW platelet distribution width, PLT-large platelet large cell ratio, ESR erythrocyte sedimentation rate, TP total protein, AST aspartate aminotransferase, ALT alanine aminotransferase, γ-GTP γ-glutamyltransferase, LDH lactate dehydrogenase, ALP alkaline phosphatase, T-BIL total bilirubin, BUN blood urea nitrogen, UA urinary acid, Cre creatinine, CRP C-reactive protein, Cre creatinine, Na sodium, Cl chloride, K potassium, Ca calcium, Glu glucose, Chol total cholesterol, CH50 50% hemolytic complement activity, RF rheumatoid factor, ASLO antistreptolysin-O, IgG immunoglobulin G, IgA immunoglobulin A, IgE immunoglobulin E, IgM immunoglobulin M, ACE angiotensin converting enzyme

## Discussions

In a recent study reported in 2021 [4], the frequency of ocular BD in Japan has shown a decrease compared to past epidemiologic survey [2, 3]. However, BD remains a relatively common form of uveitis worldwide [5–9]. Prior to the advent of anti-TNF therapy, the visual prognosis in patients with ocular BD was poor. However, with the introduction of anti-TNF therapy, the visual prognosis of ocular BD has improved drastically [12–16]. Nonetheless, some patients with ocular BD still experience recurrent episodes of ocular inflammation leading to visual impairment [12, 13]. Consequently, there is a growing consensus advocating early initiation of anti-TNF therapy [33]. However, since there are cases in which ocular inflammation does not recur without therapeutic intervention, indiscriminately initiating anti-TNF therapy in all patients with ocular BD having visual impairment may not be appropriate for both the patients and the sustainability of medical care. There thus is a need to explore biomarkers that predict the necessity of anti-TNF therapy in patients with ocular BD. Cluster analysis is a statistical method that identifies relatively homogeneous groups based on selected features and is increasingly being utilized to identify phenotypic subgroups within various diseases and to explore biomarkers for predicting clinical outcomes [17, 26, 27, 34]. Given the heterogeneity of the clinical courses of ocular BD [17, 34], unbiased cluster analysis is deemed valuable in predicting the clinical course of ocular BD and searching for predictive biomarkers of clinical outcomes.

This study utilized unsupervised hierarchical clustering analysis of routine blood test data to delineate distinct clinical phenotypes of ocular BD, revealing potential biomarkers for predicting clinical outcomes. Our findings underscore the heterogeneity of BD as a result of the diverse pathophysiological pathways and varied clinical presentations within the patient population. The four patient groups identified through our analysis (low CRP, high ACE, high ASLO, low neutrophil count) demonstrated significant differences in clinical outcomes, particularly in the frequency of ocular inflammatory attacks and the rate of TNF inhibitor initiation. Notably, patients with high percentage of monocytes in differential blood count were more likely to require treatment with TNF inhibitors, suggesting a more aggressive disease phenotype. This group also exhibited a higher rate of poor visual acuity at the first visit, indicating that early intervention may be critical for such patients. Furthermore, our study highlights the role of specific biomarkers such as neutrophil-to-lymphocyte ratio, which was associated with the frequency of ocular inflammatory attacks. These findings are consistent with previous studies suggesting that neutrophil activation plays a crucial role in the pathogenesis of BD [35, 36]

The use of machine learning algorithms enhanced the identification of key biomarkers. For example, MCHC was identified to be a predictor of the future need for TNF inhibitors. Elevated MCHC levels in BD are indicative of increased disease activity, reflecting chronic inflammation and erythrocyte morphological changes [37]. This association suggests that MCHC could serve as a valuable biomarker for monitoring BD severity and guiding therapeutic decisions. Although the results of this study show that percent monocytes is better at predicting TNF inhibitor initiation than MCHC, the application of machine learning reveals that test data which were previously unnoticed reflect the pathophysiology of the disease. Additionally, our findings suggest that conventional biomarkers used in systemic BD, such as CRP and ESR [38], are less predictive in ocular BD compared to more specific markers such as percent monocytes and MCHC. This could be due to the unique inflammatory pathways in ocular BD, which may not be as heavily dependent on the acute phase reactants typically seen in systemic BD. On the other hand, machine learning combined with logistic regression analysis revealed that percent monocytes was the most sensitive biomarker to predict TNF inhibitor initiation. Monocytes play a critical role in the pathogenesis of BD by driving chronic inflammation through the production of proinflammatory cytokines, enhancing monocyte and neutrophil adhesion and aggregation, and expressing various activation markers that exacerbate the inflammatory response [36]. Therefore, increase in percent monocytes potentially indicates an early stage of neutrophil activation. Moreover, the identification of these biomarkers provides a foundation for developing personalized medicine in BD treatment. By tailoring treatment strategies based on specific biomarker profiles, clinicians can potentially improve outcomes through more targeted therapies that address the underlying disease mechanisms specific to each patient group. For example, cases with a high neutrophil-to-lymphocyte ratio may be more prone to inflammatory attacks, indicating the need for treatment strategies that actively control inflammation. In cases with elevated monocyte levels, there may be a higher likelihood of requiring biologics in the future, necessitating continuous follow-up even if the inflammation is currently well-controlled. Furthermore, the methods used in this study could also be applicable for detecting biomarkers to predict clinical outcomes in other diseases, including other types of uveitis.

This study has several limitations that should be considered when interpreting the results. First, the retrospective design may introduce biases related to data collection and patient selection. Since the study was based on data from a single institution, the findings may not be generalizable to other populations with different demographic or clinical characteristics. For future studies, it is recommended to conduct multicenter studies that include various regions across countries to increase the sample size and improve the universality and representativeness of the study results. Second, the analysis relies solely on routine blood test data, which may not capture the full spectrum of pathophysiological changes in BD. Moreover, only 24 patients (14%) had no history of prior treatments, including topical or oral medications and supplements, at the time of initial blood sampling. This suggests that most peripheral blood test results may have been influenced by prior treatments. While these markers are useful for identifying certain trends and associations, they may not fully represent the underlying disease mechanisms. Therefore, to gain a deeper understanding of the pathophysiology of BD, it is necessary to comprehensively analyze a variety of data, including blood cytokines, proteins and metabolites, as well as cytokines, proteins and metabolites in intraocular fluids. Additionally, the biomarkers identified are not yet validated, and their predictive power and clinical relevance require further confirmation through prospective studies. Nevertheless, this study demonstrates that despite variations in treatment status and disease stage at the time of initial consultation, patient stratification is still feasible. This suggests that the identified biomarkers may reflect real-world clinical scenarios, making them potentially more applicable in routine clinical practice. Thus, while the variability of treatment status and disease activity is a limitation, it also represents a major strength of this study. Third, the sample size, although adequate for initial analyses, may limit the power to detect smaller yet clinically significant differences between patient groups. This is particularly relevant in the context of subgroup analyses, where the number of patients in each cluster might be too small to yield statistically robust conclusions. Furthermore, while the clustering technique used is powerful for identifying patterns within the data, it depends heavily on the choice of variables included in the analysis. The exclusion of potential confounders or relevant clinical parameters not routinely measured could skew the results and limit the accuracy of the predictions. Lastly, the clinical applicability of our findings is constrained by the observational nature of the study. Longitudinal studies incorporating these biomarkers in clinical trials are necessary to determine their utility in guiding treatment decisions and improving patient outcomes.

## Conclusion

In conclusion, the findings of our study provide significant insights into the complex pathophysiology of BD and underscore the potential of using routine blood test data, analyzed by advanced clustering techniques, to predict clinical courses and tailor therapeutic interventions. Further research is needed to validate these biomarkers in large, multicenter studies and to explore their utility in clinical practice.

## Supplementary Information

Below is the link to the electronic supplementary material.

Supplementary file1 (DOCX 15 KB)

## Data Availability

The data presented in this study are available on request from the corresponding author.
