# Supplementary material for: Identification of markers predicting clinical course in patients with Behcet disease by combination of machine learning and unbiased clustering analysis
Source: Graefes Arch Clin Exp Ophthalmol. 2025 May 6;263(9):2641–50. doi: 10.1007/s00417-025-06850-5 (PMC12513920; doi:10.1007/s00417-025-06850-5)
Supplement: Supplementary file 1 — Supplementary file1 (DOCX 15 KB) [file 417_2025_6850_MOESM1_ESM.docx]

| Supplemental Table S1. Previous treatment before the initial visit | |
| --- | --- |
| Treatment |  |
| Eye drops only, n (%) | 50 (28%) |
| topical steroids eye drops, n (%)* | 50 (28%) |
| topical glaucoma medications, n (%)* | 19 (11%) |
| Combination systemic therapy, n (%) | 65 (37%) |
| systemic corticosteroids, n (%)* | 20 (11%) |
| biologics, n (%)* | 15 (9%) |
| colchicine, n (%)* | 13 (7%) |
| immunosuppressive agents, n (%)* | 8 (5%) |
| acetazolamide, n (%)* | 6 (3%) |
| other systemic therapy, n (%)* | 65 (37%) |
| No treatment, n (%) | 24 (14%) |
| Unknown, n (%) | 37 (21%) |
| *With overlap Percentage was calculated with total number of eyes analyzed as denominator. | |
